# Draft Genome Sequence of *Sphingobium* sp. Strain KK22, a High-Molecular-Weight Polycyclic Aromatic Hydrocarbon-Degrading Bacterium Isolated from Cattle Pasture Soil

**DOI:** 10.1128/genomeA.00911-13

**Published:** 2013-11-07

**Authors:** Allyn H. Maeda, Shinro Nishi, Yasuhiro Ozeki, Yukari Ohta, Yuji Hatada, Robert A. Kanaly

**Affiliations:** Department of Life and Environmental System Science, Graduate School of Nanobiosciences, Yokohama City University, Kanazawa-ku, Yokohama, Japana; Japan Agency for Marine-Earth Science and Technology (JAMSTEC), Yokosuka, Japanb

## Abstract

*Sphingobium* sp. strain KK22 was isolated from a bacterial consortium that originated from cattle pasture soil from Texas. Strain KK22 grows on phenanthrene and has been shown to biotransform the high-molecular-weight (HMW) polycyclic aromatic hydrocarbon (PAH) benz[*a*]anthracene. The genome of strain KK22 was sequenced to investigate the genes involved in aromatic pollutant biotransformation.

## GENOME ANNOUNCEMENT

*Sphingobium* sp. strain KK22 is a member of a bacterial consortium that was maintained on diesel fuel and benzo[*a*]pyrene and that mineralized high-molecular-weight polycyclic aromatic hydrocarbons (HMW PAHs) (1–3). The consortium was originally recovered from soil from an active cattle pasture in the Gulf region of Texas, which had been used for this purpose for 18 years at the time of sampling (4, 5). Strain KK22 was isolated from the consortium by phenanthrene enrichment and it was shown to biotransform the HMW PAH benz[*a*]anthracene to 3-, 2- and single-aromatic ring products (6). Phylogenetic analysis of the 16S rRNA gene sequence of strain KK22 showed that it was most closely related to the alphaproteobacterium *Sphingobium fuliginis* TKP^T^ (6, 7).

The draft genome sequence of strain KK22 was determined on an Ion Torrent Personal Genome Machine (Life Technologies, Germany) (8, 9). A total of 1,255,598 reads with an average length of 257 bp were obtained, and the genomic sequence contigs were assembled *de novo* using the CLC Genomics Workbench 6.0.1 program (CLC bio, Denmark). The reads were aligned to produce 252 contigs (>500 bp) with an N_50_ of 38,414 bp and resulted in 66-fold coverage of the genome.

The total length of the draft genome is 4,916,599 bp, and the G + C content is 64.7%. The gene prediction and annotation for the assembled contigs were determined by combining results from RNAmmer 1.2, tRNA scan-SE 1.23, and the Rapid Annotations using Subsystems Technology (RAST) pipeline (10–12). The genome of strain KK22 contains one 5S rRNA gene, one 16S rRNA gene, one 23S rRNA, and 45 tRNA genes. Based on the RAST results, the draft genome includes 4,774 coding sequences (CDSs), of which 68% (3,253) were annotated based on known proteins with biological functions and 31% (1,521) were annotated as hypothetical proteins.

Sphingomonads are known for their metabolic diversity and for their roles in the biodegradation of hazardous materials, including HMW PAHs (13–15). Genes known to be involved in PAH biotransformation by sphingomonads were found to be distributed in the genome of strain KK22, including genes that code for ring-hydroxylating oxygenases. At least seven sets of putative oxygenase genes (*xylXY*, *bphA1a2a*, *bphA1b2b*, *ahdA1c2c*, *ahdA1d2d*, *ahdA1e2e*, and *bphA1f2f*) were revealed by the annotation of gene function and classification by KEGG and MetaCyc. At least one copy each of the genes that coded for ferredoxin (*bphA3*) and ferredoxin reductase (*bphA4*) was present in the genome and was localized with the first six sets of oxygenase genes. Observations such as these were reported for the relevant genomic regions of *Novosphingobium aromaticivorans* F199, *Sphingobium yanoikuyae* B1, and *Sphingobium* sp. strain P2 (16–18). Biotransformation studies are ongoing to advance our understanding of the metabolic versatility of *Sphingobium* sp. KK22.

### Nucleotide sequence accession numbers.

The draft genome sequence for *Sphingobium* sp. KK22 has been deposited in DDBJ/EMBL/Genbank under the accession no. BATN01000000. The 252 contigs have been deposited under accession no. BATN01000001 to BATN01000252.
